# Performing percutaneous nephrolithotomy under modified local anesthesia

**DOI:** 10.3389/fsurg.2022.922158

**Published:** 2022-10-11

**Authors:** Yue Yu, Jieping Hu, Wei Liu, Zhixiong Peng, Mengzhen Wang, Xiaochen Zhou, Haibo Xi

**Affiliations:** ^1^ Department of Urology, The First Affiliated Hospital of Nanchang University, Nanchang, China; ^2^ Department of Surgery, Traditional Chinese Medicine Hospital of Yichun City, Yichun, China

**Keywords:** modified local anesthesia, percutaneous nephrolithotomy (PCNL), ASA—American Society of Anesthesiologists, physical status, renal stone

## Abstract

**Objective:**

This pilot study aimed to assess the practicability and effectiveness of percutaneous nephrolithotomy (PCNL) with vacuum-assisted nephrostomy sheaths for patients under modified local anesthesia (m-LA).

**Methods:**

PCNL with a vacuum-assisted nephrostomy sheath under m-LA was performed in 83 patients between November 2020 and May 2021. An 18F or 20F ClearPetra Nephrostomy Sheath connected vacuum aspiration was used in surgery to keep low pressure in the renal pelvis. For LA, lidocaine and ropivacaine hydrochloride were 1:1 mixed and instilled under ultrasound guidance through the percutaneous nephrolithotomy channel directed toward the design calix. Demographic characteristics, stone characteristics, visual analogue scale (VAS) score, vital signs, operation time, complications, and stone clear rate were recorded and analyzed.

**Results:**

All operations were completed. The mean VAS score was 3.9 ± 1.0. The mean operation time was 55.1 ± 23.6 min. The changes for systolic blood pressure, diastolic blood pressure, and heart rate were 3 ± 21 mmHg, 1 ± 14 mmHg, and −6 ± 14 beats/min, respectively. The change for hemoglobin was −10.7 ± 10.9 g/L. The change for C-reactive protein was 5.39 ± 43.1 mg/L. The total stone-free rate was 69.9% (93.8% for simple stones and 54.9% for complex stones).

**Conclusion:**

Performing PCNL with vacuum-assisted nephrostomy sheaths under modified local anesthesia under ultrasound guidance was found to be strongly practical and effective.

## Introduction

Percutaneous nephrolithotomy (PCNL) is a minimally invasive treatment for some indicated renal stones and upper ureteral calculi. Guidelines recommend PCNL as the preferential treatment modality for all renal stones >20 mm owing to the increased stone-free rate and reduced re-treatment sessions (1). Mini tract size, vacuum suction, and other technological advances in PCNL improved the safety and effectivity, broaden the application, and enhanced recovery after surgery (2, 3). Apart from these developments, the method of anesthesia was a key point that affects the safety and recovery of patients. The American Society of Anesthesiologists (ASA) classification is a system in which patients are evaluated according to the risk of anesthesia before surgery. ASA-I represents a completely healthy patient, ASA-II represents a patient with mild systemic disease, ASA-III represents a patient with severe systemic disease that is not incapacitating, ASA-IV represents a patient with incapacitating disease that is a constant threat to life, and ASA-V represents a moribund patient who is not expected to live for 24 h with or without surgery. This system could be used to choose the type of anesthesia and to assess the tolerance of patients to various surgical manipulations such as surgical position. Staying in a prone position during PCNL could lead to some difficulties, such as those in controlling the airway and maintaining the vascular access and ventilation of patients with lung diseases (4).

Generally, PCNL is performed under general anesthesia (GA); however, comorbidities such as coronary heart disease and chronic obstructive pulmonary disease increase the risk of anesthesia (5). GA also deferred the recovery from surgery and increased the economic burden (6), increasing the research focus on local anesthesia (LA) in PCNL for decades (7–11). LA offers many advantages compared with other methods of anesthesia, as it minimally affects patients’ physiological status leading to their enhanced recovery (12). In this study, we used a vacuum-assisted nephrostomy sheath to keep low pressure in the renal pelvis and suck out the stone fragments. We modified the LA (m-LA) that was previously reported (7, 13, 14) and performed precise local anesthesia in a percutaneous nephrolithotomy channel under ultrasonic guidance; it was convinced that eventual capsular puncture site was the same spot the anesthesia needle is inserted. We performed m-LA for patients with ASA score I–III, and we also compared the practicability and effectiveness of this new procedure for different ASA score. Data on visual analogue scale (VAS) score, vital signs, operation time, complications, and stone clear rate were recorded and analyzed.

## Patients and methods

Between November 2020 and May 2021, 83 cases that met the criteria were enrolled in the study. Inclusion criteria were (1) renal stone larger than 2 cm, (2) upper ureteral calculi larger than 1 cm, and (3) the patient was willing to receive local anesthesia. Exclusion criteria were (1) concomitant middle or lower ureteral calculi, (2) stone secondary to ureteral stricture, (3) second-stage operation, (4) BMI >30 kg/m^2^, and (5) local anesthesia drug allergies. Patients routinely received urinalysis, urine culture, routine blood test, serum creatinine, and electrolyte test. Upper urinary calculi were diagnosed by ultrasound, kidney, ureter, bladder x-ray, and CT scans. Simple stones refer to single renal or ureteral stones, and complex stones refer to multiple stones, except in patients with anatomical abnormalities. In addition, complex stone included staghorn calculi.

Antibiotics including latamoxef or etimicin were administered to patients 30 min before the operation. The patient was in the prone position, and precise local anesthesia in the percutaneous nephrolithotomy channel was performed under ultrasonic guidance; the key points were (1) 1% lidocaine 10 ml and 1% ropivacaine hydrochloride 10 ml were mixed for local injection, lidocaine had rapid onset, but short effectiveness, ropivacaine had a long-term effect, and the mixture giving full play of merits. (2) A 20GA 1.88IN venous indwelling needle [Singapore Becton Dickinson Medical (S) Pte Limited] with a 20 ml syringe was used for drugs injection, with the fine needle relieving the injection pain. (3) The injection of anesthetic was along the percutaneous nephrolithotomy channel under ultrasonic guidance (Noblus, Hitachi, Ltd.), and anesthesia reached the renal fascia. (4) Using an 18F or 20F ClearPetra Nephrostomy Sheath (Cat No: 90121617, 90121817, Well Lead Medical Co., Ltd) in surgery to keep low pressure in the renal pelvis can avoid distending pain in the kidney. (5) No ureteral catheter was inserted in any of the patients; it can avoid the pain caused by inserting a ureteral catheter, and furosemide (20 mg) would be given by injection into a vein to form artificial hydronephrosis, which was convenient for puncture.

**Figure 1:**
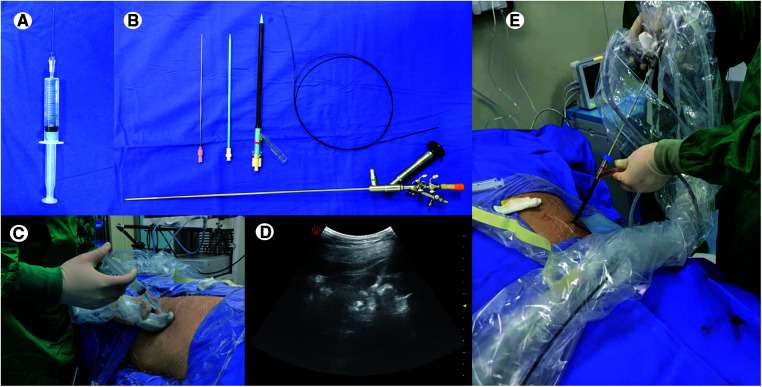
Main instruments and operation procedures of percutaneous nephrolithotomy for modified local anesthesia. (**A**) 1% lidocaine 10 ml and ropivacaine hydrochloride 10 ml were mixed for local injection, and a 20GA 1.88IN venous indwelling needle [Singapore Becton Dickinson Medical (S) Pte Limited] with a 20 ml syringe was used for drug injection. (**B**) An 18-gauge needle was used for percutaneous punctures, a 12F or 14F fascial dilator was applied for dilation, and an ureteroscope (8/9.8F) was for laser lithotripsy. (**C,D**). The injection of anesthetic was along the percutaneous nephrolithotomy channel under ultrasonic guidance. (**E**) An 18F or 20F ClearPetra Nephrostomy Sheath was used for the establishment of a percutaneous renal channel; the lateral channel of the sheath connected with negative suction ensures intrapelvic low pressure and stone fragments were easy to flush out.

Other procedures were routinely performed as previously reported (8), and some were modified accordingly (15, 16). After anesthesia, percutaneous punctures using an 18-gauge needle (Cat No: G01377, Cook Medical) were guided by ultrasound in the channel. After removing the needle core, the guidewire was inserted into the collecting system through the needle, the skin was incised, and the needle was removed with recording the depth. A 12F or 14F fascial dilator was applied for dilation and entered the depth of the earlier record. The ureteroscope (8/9.8F) was moved to the renal pelvis following the guidewire to confirm channel passage through the renal papilla. An 18F or 20F ClearPetra Nephrostomy Sheath was subsequently placed into the channel. The stone was fragmented by a high-power holmium laser (2.5 J × 35 Hz); fragments were pushed out by an endoscopic pulsed perfusion pump and vacuum aspiration (Figure 1). Finally, a 4.7F or 6F double-J stent was inserted in the ureter; a nephrostomy tube was used depending on inflammation, residual stones, and hemorrhage.

The demographic characteristics that included sex, age, size of the stone, location of the stone (single stone in renal or ureter was recorded as simple, others were complex), and comorbidity (chronic obstructive pulmonary disease, cardiovascular disease, etc.) of the patients were recorded. The surgery data that included the tract number, duration of the operation, systolic blood pressure (SBP), heart rate (HR), and VAS scores were evaluated. Postoperative evaluations were drop in hemoglobin, fever, perirenal hematoma, pleural effusion, residual stone, length of hospital stay, medical cost, and Barthel index. An x-ray of KUB was taken after one day after surgery. We defined stone-free rate as the radiological absence of stone in asymptomatic patients with residual stone fragments <3 mm (17). Blood pressure and heart rate were recorded before surgery, and the highest and lowest values were recorded during the operation.

## Results

### The demographic characteristic of patients

The characteristics of included patients are listed in Table 1. A total of 52 men and 31 women received m-LA for PCNL. The mean age was 53.6 ± 13.9 years. Nine patients had hypertension, 6 had diabetes, 2 had paraplegia, 10 had coronary heart disease, and 7 had poor pulmonary function. Preoperative SBP was 133 ± 20 mmHg, and diastolic blood pressure (DBP) was 83 ± 12 mmHg, heart rate was 79 ± 11 beats/min, and the body mass index was 23.4 ± 4 kg/m^2^. Patients with ASA score I–III were 51, 17, and 15, respectively. The mean hemoglobin was 130 ± 20 g/L, and the mean C-reactive protein (CRP) was 15.3 ± 32.9 mg/L. A total of 32 cases were simple stones, and 51 cases were complex stones. The size of the stone was 19.4 ± 8.1 mm.

**Table 1:** Characteristic of the included patients.

Parameters	Mean (standard deviation)	No.
Sex (men/women)		
Men		52
Women		31
Age (year)	53.6 (13.9)	
Preoperative blood pressure (mmHg)		
Systolic pressure	133 (20)	
Diastolic pressure	83 (12)	
Preoperative heart rate	79 (11)	
Body mass index (kg/m^2^)	23.4 (4.0)	
ASA score		
I		51
II		17
III		15
Type of stone		
Simple		32
Complex		51
Size of stone (mm)	19.4 (8.1)	
Hemoglobin (g/L)	130 (20)	
C-reactive protein (mg/L)	15.3 (32.9)	

ASA, American Society of Anesthesiologists.

### The main clinical results

The mean VAS score was 3.9 ± 1.0. The mean operation time was 55.1 ± 23.6 min. The postoperative hospital stay was 4.1 ± 1.2 days. The changes for SBP, DBP, and heart rate were 3 ± 21 mmHg, 1 ± 14 mmHg, and −6 ± 14 beats/min, respectively. The change for hemoglobin was −10.7 ± 10.9 g/L. The change for C-reactive protein was 5.39 ± 43.1 mg/L. There were 64, 17, and 2 patients who received 1, 2, and 3 tracts, respectively. It was noted that 55, 26, and 2 patients retained 0, 1, and 2 nephrostomy tubes. One patient received a blood transfusion and renal arterial embolization, and there was no Clavien grade IV–V complications. The total stone-free rate was 69.9%, which was 93.8% for simple stones and 54.9% for complex stones. The main clinical results of the patients are listed in Table 2.

**Table 2:** The main clinical results of the patients.

Parameters	Mean (standard deviation)	No.
Duration of the operation (min)	55.1 (23.6)	
Tract number		
1		64
2		17
3		2
Postoperative blood pressure (mmHg)		
Systolic pressure	136 (17)	
Diastolic pressure	84 (10)	
Change in blood pressure (mmHg)		
Systolic pressure	3 (21)	
Diastolic pressure	1 (14)	
Postoperative heart rate	74 (12)	
Change in heart rate	−6 (14)	
Hemoglobin (g/L)	120 (19)	
C-reactive protein (mg/L)	20 (34)	
Change in hemoglobin (g/L)	−10.7 (10.9)	
Change in C-reactive protein (mg/L)	5.39 (43.1)	
Complications		
Transient fever		1
Renal collecting system injury		0
Perirenal hematoma		1
Blood transfusion		1
Renal arterial embolization		1
Pleural injury requiring drainage		0
Clavien grade IV–V		0
Stone-free rate	69.90%	
Nephrostomy tube		
0		55
1		26
2		2
Postoperative hospital stay (days)	4.1 (1.2)	
VAS score	3.9 (1.0)	

VAS, visual analogue scale.

### Subgroup analysis according to ASA status

Subgroup analysis was performed according to ASA status; the patient's perioperative status is recorded in Table 3. Sex, BMI, type of stone, VAS score, stone-free rate, preoperative SBP, DBP, hemoglobin, CRP, duration, tract number, and postoperative SBP, DBP, HR, and CRP showed, no significant differences among the ASA-I–III patients. Patients were younger in the ASA I group, and postoperative hemoglobin was higher. However, hemoglobin change showed no significant difference.

**Table 3:** The characteristic of patients according to ASA score.

Parameters	ASA			*P* value
	I	II	III	
VAS score	3.9 (1.1)	3.9 (0.9)	3.6 (0.9)	0.514
Sex				0.082
Men	28	11	13	
Women	23	6	2	
BMI	24.1 (3.9)	21.6 (4.1)	23.1 (3.8)	0.082
Type of stone				0.361
Simple	22	4	6	
Complex	29	13	9	
Size of stone	19.1 (7.3)	21.1 (10.0)	18.4 (8.3)	0.597
Stone-free rate	38/51	10/17	10/15	0.464
Age	49.4 (11.2)	60.7 (14.5)	59.7 (16.5)	0.002
Preoperative				
SBP (mmHg)	129 (16.6)	139.8 (27.6)	139.9 (17.9)	0.055
DBP (mmHg)	82.6 (10.2)	82.6 (15.5)	84.5 (13.6)	0.862
Heart rate	77.2 (9.1)	85.9 (12.2)	80.1 (14.5)	0.019
Hemoglobin (g/l)	133.9 (19.1)	122.2 (23.6)	127.1 (17.8)	0.092
C-reactive protein (mg/l)	12.9 (32.7)	28.7 (41.5)	8.0 (16.5)	0.149
Duration	56.4 (27.2)	55.4 (15.6)	50.3 (17.5)	0.677
Tract number				0.983
1	40	13	11	
2	9	4	4	
3	2	0	0	
Postoperative				
Systolic blood pressure (mmHg)	133.6 (14.1)	141 (18.8)	138.5 (22.3)	0.237
Diastolic blood pressure (mmHg)	85.6 (9.1)	82.3 (7.8)	82.3 (14.7)	0.349
HR	71.4 (11.8)	77.8 (10.1)	77.8 (12.1)	0.052
Hb (g/L)	124.7 (16.9)	113.4 (20.3)	112.9 (18.9)	0.028
CRP (mg/L)	21.1 (38.3)	17.9 (14.9)	18.2 (36.9)	0.934
Postoperative hospital stay	4.0 (1.3)	4.3 (0.9)	4.2 (1.3)	0.689
SBP change	4.5 (19.4)	1.2 (21.5)	−1.3 (27.3)	0.613
DBP change	3.0 (11.8)	−0.3 (12.0)	−2.2 (21.4)	0.387
HR change	−5.8 (13.4)	−8.1 (11.1)	−2.3 (20.6)	0.532
Hb change	−9.9 (9.7)	−10.5 (15.8)	−14.0 (8.4)	0.445
CRP change	7.7 (44.9)	−10.5 (40.7)	14.4 (36.8)	0.251

ASA, American Society of Anesthesiologists; VAS, visual analogue scale; BMI, body mass index; SBP, systolic blood pressure; DBP, diastolic blood pressure; HR, heart rate; Hb, hemoglobin; CRP, C-reactive protein.

## Discussion

Local anesthesia broadens the indications for PCNL. It hedged risks caused by deformity (chest and spine), and poor heart and lung function; meanwhile, the renal function may be maximum preserved (5, 14). Other advantages were obvious, such as reduced medical cost, short hospitalization time, less inﬂuence on patients’ physiological functions, and faster recovery (12, 13). In this study, we modified the LA to perform precise local anesthesia, through which we wanted to control pain throughout the surgery.

Previous reports about LA in PCNL were improvable (8, 10, 18, 19). The characteristics of LA are listed in Table 4, and progresses in our study were noticed in the methods. First, patients received premedication such as pethidine or promethazine 0.5–1 h before surgery as in a previous study; we did not apply these drugs to avoid possible side effects. Second, the local anesthetic was lidocaine or ropivacaine in a previous study, which cannot reconcile rapid onset and long duration; the mixture combined the advantages of both. Third, we applied a ClearPetra Nephrostomy Sheath rather than a Peel-Away Sheath; the new sheath could effectively control intrarenal pressure and reduce pain in surgery. Fourth, we performed precise LA (i.e., injection of anesthetics in percutaneous nephrolithotomy channel under ultrasonic guidance), which maximized the anesthetic effect. Finally, for some patients with none or mild hydronephrosis, the usage of furosemide can avoid retrograde ureteral catheterization. Thus, we modified LA and assessed its practicability and effectiveness.

**Table 4:** The characteristics of LA in previous studies.

Study	Haleblian (2007)	Li (2013)	Ecke (2017)	Wang (2019)
Region	European	Asian	European	Asian
Premedication	Pethidine HCl (100 mg) and diazepam (0.1 mg/kg orally)	Pethidine premedication (75–100 mg) and Phenergan (25 mg)	Dormicum (7.5 mg)	50–75 mg pethidine hydrochloride and 25 mg promethazine hydrochloride
Antibiotic prophylaxis	300 mg dose of netilmicin	NA	NA	Cefazolin or cefotiam 1 g in 0.9% saline
Hypodermic needle	NA	23-gauge spinal needle	22-gauge spinal needle	NA
Anesthesia drug	2% lignocaine	1% lidocaine	Ropivacaine	1% lidocaine
Guidance	Ultrasound	Ultrasound	X-ray	Ultrasound
Tract size	27F	16–30F	28F	22F
Lithotripter	Ballistic lithotripter	Pneumatic lithotripsy system and/or holmium laser	Sonotrode system	EMS V Lithoclast Master
VAS score	3.6 (1.3–6.9)	3.62	NA	6.0 ± 2.0

LA, local anesthesia; VAS, visual analogue scale.

In our study, m-LA in PCNL achieved satisfactory results. All operations were completed successfully, and none of m-LA was converted to GA; we continuously communicated with patients during the operation to monitor pain, and local anesthetics can be added for analgesia during the operation. For some stones with large angles, the stone clearing rate may be sacrificed to complete the operation. No serious cardiovascular incident occurred, no death occurred, and postoperative rehabilitation was satisfactory. The pain was evaluated using a VAS score. The mean VAS score was 3.9 ± 1.0, and the pain levels during the operation were mild and could be tolerated, which was similar to previous reports (8, 10). Our method had advantages compared with other research studies (12). The vital signs were not reported by previous studies. Our research recorded the heart rate and blood pressure during surgery, and results showed that the indexes were changed within a limited range, indicating m-LA had a minor impact on hemodynamics.

Other parameters also evaluated the effect of surgery. Operation times were 55.1 ± 23.6 min. One patient felt discomfort and discontinued surgery because of keeping a long-time prone position; due to the lack of preoperative preparation of jejunitas, the patient was not converted to GA. He received a II-stage operation under m-LA on the second day, and the stone was removed. The total stone-free rate was 69.9%, which was 93.8% for simple stones and 54.9% for complex stones. It seems that the total stone-free rate was inferior to what was previously reported (20); the reason may be complex stones were the majority (61.4%) in this research. Complications were seldom, Clavien grade IV–V complications, and injury in the renal collecting system did not occur in our group; however, transfusion was needed for one patient. The mean hemoglobin change was −10.7 ± 10.9 g/L. The important skill to reduce complications relied on precise ultrasonic guidance, not only performing m-LA but also choosing the puncture site, to avoid bleeding and organ injury. Owing to the complication reduction and rapid recovery, hospital stay was only 4.1 ± 1.2 days.

Our study did not simply duplicate the work of our predecessors, and the key points for our study are listed as follows. First, although obesity was not a contraindication, obese patients were not encouraged for PCNL under m-LA, although data indicated that BMI was not associated with VAS score (the Pearson coefficient was 0.113, *P *= 0.309). The swing of the sheath was restricted and increased pain. Second, patients with renal stones in the polar calix were not included for the same reason. Third, we recommended two-step puncture techniques for PCNL (15). It reduced pain caused by re-dilation procedures and ensured the channel through renal papillae. Fourth, ClearPetra Nephrostomy Sheath was important for reducing intrarenal pressure and distending pain because it was of Y type to connect with vacuum aspiration, and the use of ordinary sheath should control the pump to limit the intrarenal pressure. Fifth, this was the first time to analyze the vital signs and the changes in PCNL under m-LA, and we found that m-LA did not significantly alter the blood pressure and heart rate. Sixth, previous studies did not assess the practicability and effectiveness of LA for patients according to ASA score, and our research indicated that m-LA was safe for ASA-III patients.

The main limitation of our study was the limited population. Some previous reports about LA contained hundreds or thousands of patients (8, 9), and a larger number would be needed to verify our procedures. In addition, the results were concluded from one single center, and we planned to extend the technique to assess the practicability and effectiveness. Nevertheless, we provided an innovation for percutaneous nephrolithotripsy under local anesthesia.

## Conclusion

Performing PCNL with a vacuum-assisted nephrostomy sheath under modified local anesthesia under ultrasound guidance was found to be strongly practical and effective.

## Data Availability

The raw data supporting the conclusions of this article will be made available by the authors, without undue reservation.
